# Working with(out) a net: improvisational theater and enhanced well-being

**DOI:** 10.3389/fpsyg.2013.00929

**Published:** 2013-12-10

**Authors:** Gordon Bermant

**Affiliations:** Department of Psychology, University of PennsylvaniaPhiladelphia, PA, USA

**Keywords:** improv, theater, mindfulness, positive psychology, person-centered therapy, well-being, embodiment, enaction

Improvisational theater (improv) fits well into an academic definition of improvisation: “the process and product of creativity occurring simultaneously” (Lewis and Lovatt, 2013). The simultaneity in improv comes from the lack of scripting: players appear on stage, ask the audience for a suggestion, for example, “any object smaller than a breadbox,” and begin a frolic of their own that often lasts about 30 min. This article highlights several parallels between practices in improv and practices in several domains of applied psychology: body awareness and mindfulness, positive psychology interventions, and person-centered psychotherapy. What accounts for these parallels? Here I conclude that both improv and applied psychology practices aim to increase personal awareness, interpersonal attentiveness, and trust among members of the ensemble.

The metaphor of “working without a net” certainly seems to apply to improv. For example, a respected improv manual says, “True improvisation is getting on-stage and performing without any preparation or planning… Strictly speaking, improvisation is making it up as you go along” (Halpern et al., 1994). Nervous anticipation of performing without a memorized script is the felt sense of working without a net. But there is more to the story than that; we return to a reversal of the metaphor at the end of the article.

Spontaneity, like luck, favors the well-prepared. The virtuoso pianist who improvises brilliantly may be one who practiced more than 10,000 h as a youngster, on fire with a “rage to master” (Ericsson et al., 1993; Winner, 1996). Or perhaps not; there is dispute about the relationship between talent and practice at the high-end of performance skill in some fields (Epstein, 2013). Nevertheless, all forms of improvisation and competitive performance require basics to be learned and practiced until they become “second nature.” Improv actors practice their form repeatedly but don't repeat or rehearse the content of their scenes. The concept of spontaneity needs to be considered in this context: spontaneous creative content is grounded in improv fundamentals that are practiced repeatedly. It is form, not content, that is rehearsed. Many of these practices, also called “games,” are also used by actors training for scripted theater (e.g., Stanislavski, 1989).

Our inquiry is whether there is a positive relationship between improv practice and well-being in other life domains. There is a good reason to think so: important theatrical skills are represented, with different names, in the methods of body awareness and other meditations, applied positive psychology, and psychotherapy. If particular practices in these domains are psychologically healthy, then practicing them through improv might also increase well-being. Finally, because improv is unavoidably social, performers are always embedded in the social milieux of improv communities. There are transitions from classroom and small stage to what is after all “but a stage”: life itself.

## The relationship of improv to embodiment

Embodiment theory posits that cognition is an integration of information across sensory modalities into representations that retain some form of modal identifiability and can be retrieved to support perception, introspection, and action (Barsalou, 2008). Action is taken to have reflex, locomotive, instrumental, and expressive components (Gallagher, 2005). For the actor, turning locomotion and instrumental acts into gestural expressions is a skill to be developed. Every posture and motion of the actor's body should communicate the mood and intention of the character. Unsurprisingly, therefore, acting manuals stress the importance of paying close attention to sensations, being sensitive to the body's position in space, and mastering the silent projection of meaning from stage to audience (Hagen, 1973; Strasberg, 1988; Stanislavski, 1989; Chekov, 1991; Halpern et al., 1994; Spolin, 1999; Adler, 2000).

In both natural ontogeny and adult learning, sensorimotor couplings are the basis of intelligence and skill (Smith and Gasser, 2005; May, 2011). Bringing connections to consciousness can serve their skilled deployment, for example in theatrical and musical performance. Several well-being practices emphasize bodily awareness, and at least one makes a direct connection to theatrical and vocal performances: the *Alexander Technique* is named for an Australian actor who worked out the fundamentals of postural improvement and self-control to solve a problem of voice failure on stage (McEvenue, 2002; Nicholls, 2008). McEvenue specializes in applying Alexander principles to stage work. He lists seven core concepts: recognizing habits of movement, inhibiting unwanted movement habits, controlling movement by lengthening the spine, gaining conscious control of the details of successive movements, learning to distinguish accurate from faulty kinesthesis, attending to the successful accomplishment of movement while avoiding excessive concern with achieving the movement's purpose, and becoming aware of “moving with ease,” following the adage “less is more.” While Alexander methods have been subject to some traditional clinical evaluations (Nicholls, 2008), effectiveness on stage is not easily evaluated. Still, a body awareness technique that started as a practical procedure to improve theatrical performance has found significantly broader applicability.

Another example of increasing body awareness is in the “body scan” exercise that is a standard part of modern mindfulness teaching, for example mindfulness-based stress reduction (Kabat-Zinn, 1990). The therapeutic effort is directed at becoming aware of the body, in parts and together, non-judgmentally. There is substantial overlap here with “loosening up” in respect to the body and body image.

Acting of all sorts is saturated with the principles of embodiment. Improvisation in particular is a form of acting that exemplifies the meaning of *enaction*.

## The relationships of improv to enaction and musical improvisation

The primary metaphor evoking the concept of enaction is *laying down a path in walking*. Varela et al. (1991) chose this as the “guiding metaphor” to express their world-view that individuals are causal agents in the lived world whose every move changes that world just as the individual is changed by the world. Musical and theatrical improvisation exemplify the principle: each step in improvisation changes the context in which the subsequent steps will be taken. The boundaries of shifting contexts are limited only by the skills, courage, and mutual trust of the participating individuals as an ensemble.

Appreciating the similarities between musical and theatrical improvisation is possible scientifically and aesthetically. Sawyer (1993) developed a detailed theory of group creativity based on observations and interviews of jazz and theater improvisers. Among other comparisons, he emphasized that in both domains, successful improvisation depends on prior mastery of the conventions of that domain. There are gestures and “beats” in theater improv which are analogs to the “standards” of the jazz repertoire. Artists who come together to improvise expect each other to be masters of the conventional repertoire of their shared domain.

A stunning example of theatrical improvisation in a highly-controlled musical setting can be appreciated in the solo work of Zakir Hussain, the master Indian tabla drum player. Each stroke on a tabla has an associated sound (*bol*). Skilled players can vocalize the *bols* at high speed, “reading out the score” as it were, either before or after they play it on the drum. Zakir accomplishes the vocalization *conversationally*, addressing the audience with cadence and intonation as if the bols were the syllables of a natural language. The style of this presentation is, to a large degree, up to him; he is free to improvise it, though the musical script is fixed. The performance must be seen and heard to be fully appreciated, for example at http://www.youtube.com/watch?v=ZtRPB8xHP8M.

## The “Magic if,” “Fake it until you make it,” and “Act well to be well”

“*Every movement you make on the stage, every word you speak, is the result of the right life of your imagination*” (Stanislavski, 1989). In improv, “right imagination,” shared among the players, is all that there is. Psychologically, we can distinguish two levels of imagination: first, imagine *yourself* in the circumstances of another character; second, imagine *that character in that situation,* given what you have been given or imagine about the character's personality, physical nature, and moral stature. Many great acting teachers have described this distinction in various terms and different emphases (Hagen, 1973; Strasberg, 1988; Stanislavski, 1989; Chekov, 1991; Adler, 2000). All pointed to the “magic if” that allows the actor to inhabit her character authentically. Acting, as pretending, allows one to be as one is otherwise not; with correct practice, the role becomes easier to perform. It is, at bottom, *complete acceptance of a counterfactual* presented by the script or the initiation of an improv partner.

The casual phrase “fake it till you make it” is used frequently in meetings of Alcoholics Anonymous and by motivational speakers (e.g., http://www.addictiondirections.com/fake-it-til-you-make-it/; http://bestmotivationalspeaker.com/). The relationship to the “magic if” of acting is clear enough. More recently, a research program pursuing the therapeutic and character-building effects of such role-playing has been conceptualized with the title “act well to be well” (Blackie et al., in press). So there is a convergence among acting/improvising training, practical wisdom in the recovery community, and empirical research in personality psychology.

## Group mind, mind meld, and group flow

A salient feature of improv is the extraordinary cooperation that sometimes arises in a scene; when it does, it is memorable to the players and audience alike. Called “group mind” in the teaching manuals, it is characterized as a subordination of ego to the unconscious tendencies of self and other players in the scene (Halpern et al., 1994). Improv teachers have developed exercises encouraging this practice, for example “mind meld,” in which two players trade free associations until they arrive at the same word simultaneously. (http://wiki.improvresourcecenter.com/index.php?title=Mind_Meld).

The pace and intensity of the group mind experience takes the moment beyond reflective self-consciousness. Improv group mind shares this characteristic with the description of “flow” originated by Mihaly Csikszentmihalyi (Nakamura and Csikszentmihalyi, 2011). There is a great sense of camaraderie and mutual respect emerging after an improv performance “in flow.” Sawyer (1993) says of group flow “everything seems to come naturally; the performers are in interactional synchrony. In this state, each of the group members can even feel as if they are able to anticipate what their fellow performers will do before they do it.” Sawyer used examples from musical and theatrical improvisation to argue that group flow is a socially emergent property that differs from individual flow: the group and the audience may recognize it without all the performers reporting the occasion as special. Also, in both forms of improvisation, some pairs or trios “meld” more consistently and completely than others; musicians and actors have preferred partners with whom group flow emerges particularly well.

## “Yes, &… ” and unconditional positive regard

The central tenet of improv is the unambiguous and complete support of performing partners for each other. In creating and sustaining a scene, the slogan for this openness to the other is *Yes*, &… Halpern et al. (1994) write that “Yes, &… is the most important rule in improv… [It] means that whenever two actors are on stage, they agree with each other to the Nth degree.” Importantly, they distinguish the destructive effects of denying the offer of the partner from the acceptance of an offer of *conflict*, in which players implicitly agree to disagree and so argue or quibble in ways that promote the comedy of the situation. The distinction between denying an offer and consenting to conflict can be difficult for an improv pair to learn and honor.

The desired attitude between players bears a family resemblance to the concept of *unconditional positive regard* (UPR), which originated in Rogers' person-centered-psychotherapy and has since been theorized in detail (Iberg, 2001). There is at least a surface-level resemblance between UPR and the desired cognitive/emotional stance of the improviser toward her partners. It would take a bit of work to go below the surface to find deeper connections, but some of Iberg's analyses suggest that such connections exist. For example, he points to UPR as *activity*, i.e., close and positive “regarding,” as active engagement with the other, accepting the other's comments without condition. This seems to be exactly what is desired between the members of an improv pair while on stage.

If at least some of what is contained in UPR as a therapeutic stance is also contained in the Yes &… precept of improv, then perhaps improv might become another way to foster UPR growth among therapists.

## Working with an enacted net

Improv plays out in social context. It can be frightening to anticipate going on stage to make it up as you go along. This is the felt sense working without a net. But there is a source of support in improv that can alleviate the fear of failure. It is the realization that that my only obligation on stage is to my scene partner, whose only obligation is to me. In the terms already introduced, there is reciprocity of UPR and Yes &… in every exchange. If all play authentically to each other, fear of failure loses its sting—a net of support is constructed from the openness, trust, and acceptance expressed within the ensemble. Individual vulnerability creates collective strength. In this setting, failure is not a meaningful concept. To experience this in any context is a stimulus to enact it in others. The contexts will differ markedly, but the cognitive-affective matrix of individuals and ensembles can become equally wholesome.
